# Waved with open eyelids 2 (*woe2*) is a novel spontaneous mouse mutation in the protein phosphatase 1, regulatory (inhibitor) subunit 13 like (*Ppp1r13l)* gene

**DOI:** 10.1186/1471-2156-13-76

**Published:** 2012-08-28

**Authors:** Joseph Toonen, Lina Liang, Duska J Sidjanin

**Affiliations:** 1 Department of Cell Biology, Neurobiology and Anatomy, Medical College of Wisconsin, 8701 Watertown Plank Rd, Milwaukee, WI, 53226, USA; 2 Human and Molecular Genetics Center, Medical College of Wisconsin, Milwaukee, WI, USA

**Keywords:** EOB, Eyelid, Embryonic, Meibomian, Mapping, Microsatellite, Locus, Mutation, Deletion

## Abstract

**Background:**

Waved with open eyelids 2 (*woe2*) is a novel autosomal recessive mouse mutation that arose spontaneously in our animal facility. Upon initial evaluation, mutant mice exhibited eyelids open at birth (EOB) and wavy fur phenotypes. The goals of this study were to phenotypically characterize the *woe2* mice and to identify the gene harboring the mutation responsible for the *woe2* phenotype.

**Results:**

Histological analysis of *woe2* embryos identified the failure of embryonic eyelid closure. Clinical and histological analysis of *woe2* adult eyes identified severe corneal opacities, abnormalities of the anterior segment of the eye, and the absence of meibomian glands. Abnormalities in the fur texture and the absence of meibomian glands prompted us to evaluate other epidermal appendages*:* skin, teeth, and nails--as well as lacrimal, mammary, salivary, sebaceous and sweat glands. No obvious morphological differences between WT and *woe2* mice were identified in these tissues. However, the analysis of *woe2* identified cardiac abnormalities. Positional cloning of the *woe2* locus identified a 1308 bp deletion in the *Ppp1r13l* gene. The deletion resulted in an aberrant *Ppp1r13l*^*Δexon9-11*^ transcript that lacks exons 9, 10 and 11 resulting in a premature stop and a loss of 223 amino acids from the C-terminal end of the putative mutant PPP1R13L protein. Immunohistological analysis during eye development identified expression of PPP1R13L in the palpebral epidermis, palpebral and bulbar conjunctiva, corneal epithelium and meibomian glands.

**Conclusions:**

The *woe2* mouse harbors a novel deletion within the *Ppp1r13l* gene, likely resulting in a complete loss of PPP1R13L function. Results from this study provide evidence that PPP1R13L has an essential role in embryonic eyelid closure as well in development of meibomian glands and the anterior segment of the eye. The *woe2* mice are a useful model for investigation of the role of PPP1R13L, especially during ocular and eyelid development.

## Background

As a part of mammalian ocular development the eyelids form, grow over the cornea, meet, and temporarily fuse [1]. The closed eyelids serve as a protective barrier preventing premature exposure of developing ocular structures to the environment. Once conjunctival and corneal epithelia reach their maturity, the eyelid junction begins to break down and the eyelids reopen [2]. Whether different mammalian species are born with the eyelids open or closed is determined by the stage of the ocular development specific for the species at the time of birth [1]. In mice, eyelid formation is initiated at embryonic day 11.5 (E11.5) by invagination of the dorsal and ventral periocular ectoderm resulting in the formation of the primitive eyelid [1,3]. By E15.5, the leading edges of the eyelid form at the tip of the primitive eyelids and start to migrate towards each other. By E16.5, the eyelids meet and form a junction and by E17 embryonic eyelid closure is fully completed [1,3]. Mice are born with their eyelids closed and they remain closed until postnatal days 10 to 12 (P10-12) at which time they reopen [2]. When eyelids fail to close properly before birth, the result is often severe corneal inflammation, defects in the anterior segment and blindness [3,4]. Forward and reverse genetic approaches have identified genes essential for embryonic eyelid closure that, when mutated, lead to the “eyelids-open-at-birth” (EOB) phenotype. Given that mice are born with their eyelids closed, the EOB phenotype is easily identified by observation of the eyelids of newborn pups. EOB phenotypes have been identified to be associated with many genotypes; in fact, Mouse Genome Informatics (MGI) (http://www.informatics.jax.org/) lists 138 genotypes associated with the EOB phenotype indicating numerous genes and molecular pathways associated with this process.

A subgroup of mutant mice with the EOB phenotype also exhibit wavy fur. Molecular and genetic analyses showed that the majority of the mice with EOB and wavy fur phenotypes have defects in epidermal growth factor receptor (EGFR) signaling. Specifically, mice with mutations in the transforming growth factor alpha (*Tgfa*) [5,6], a disintegrin and metallopeptidase domain 17 (*Adam17) *[7-9] and *Egfr* which encodes the EGFR receptor [10-14] all exhibit EOB and wavy fur phenotypes. EGFR signaling plays an essential role in regulating the eyelid leading edge migration through activation of the EGFR-ERK signaling cascade [15]. Interestingly, another mouse mutant termed *waved 3* (*wa3*) also exhibits the EOB and wavy fur phenotypes similar to those observed in mice with EGFR signaling defects [16]. The *wa3* mice have a mutation in *Ppp1r13l *[16]*,* a gene that belongs to the Apoptosis Stimulating Proteins of p53 (ASPP) family of proteins. Although PPP1R13L is a highly conserved protein from *C. elegans* to human [17] the role of PPP1R13L remains poorly understood. It has been shown that PPP1R13L acts as a regulator of p53-mediated apoptosis [17] and as a regulator of the NF-кB subunit p65-RelA gene expression [18]. Recently, it was also shown that PPP1R13L, via its regulation of p63, is a key regulator of epithelial homeostasis [19] and epithelial stratification [20].

Here we report a novel autosomal recessive mouse mutation that arose spontaneously in our mouse colony. Initial observations showed that the mutant mice exhibit EOB and wavy fur phenotypes. The identified phenotypes observed in the mutant mice resemble those in *waved with open eyelids (woe*) mice carrying a hypermorphic mutation in *Adam17,* previously studied in our lab [8]. Thus, we termed the newly identified mutant mice *waved with open eyelids 2* (*woe2*). A detailed examination of *woe2* phenotypes revealed that a defect in embryonic eyelid closure is responsible for the *woe2* EOB phenotype observed at birth. Additional ocular phenotypes in *woe2* mice include severe corneal opacities, defects in the structures of the anterior segment of the eye, and the absence of the meibomian glands. In addition to ocular and wavy fur phenotypes, *woe2* mice also exhibited severe cardiac defects. Genetic analysis showed that the *woe2* phenotypes are due to a 1308 bp deletion in the *Pppr13l* gene. The identified deletion results in aberrantly spliced *Ppp1r13l* transcript and a putative truncated PPP1R13L protein lacking C-terminal functional domains. These findings uncover previously unidentified roles for PPP1R13L during eyelid and ocular development.

## Methods

### Mice

The *woe2* mutation arose spontaneously on a mixed C57BL/6X129/SvJ background. The *woe2* locus was maintained by brother-sister breedings. The C3A.BLiA-*Pde6b+*/J strain, which is a *Pde6b(+)* strain of C3H/HeJ (http://jaxmice.jax.org/strain/001912.html), and C57BL/6J were obtained from the Jackson Laboratory (Bar Harbor, ME). All strains exhibited normal breeding patterns and litter sizes. The treatment and use of all animals in this study was compliant with all protocols and provisions approved by the Institutional Animal Care and Use Committee (IACUC) at the Medical College of Wisconsin.

### Clinical evaluation, histology and electron microscopy

For clinical analysis, mouse eyes were examined with a Topcon SL-D8Z slit lamp biomicroscope, following mydriasis with 1% Atropine Sulfate (Bausch & Lomb). The eyes were imaged with a Nikon SLR-based Photo Slit Lamp imaging system as previously described [21]. For tissue analysis, E0.5 was defined as the morning of the day that a vaginal plug was first observed in a female. Embryonic and postnatal tissues were collected and fixed in either Zinc-formalin, Davidson’s solution, or 4% paraformaldehyde, then embedded in paraffin and sectioned to 4 μm thickness and stained with H&E using standard procedures as previously described [8]. For scanning electron microscopy (SEM), E15.5 and E16.5 wild-type and *woe2* embryo heads were collected, fixed in 2% glutaraldehyde in 0.1 M sodium cacodylate buffer, rinsed in buffer and dehydrated in ethanol. The samples were then critical-point dried in a Bal-tec CPD050, gold sputter coated in a Denton Desk II and viewed in a FEI XL30 SEM.

### Immunohistochemistry

Antigen retrieval was performed in 1x citrate Buffer (Invitrogen) warmed to 95°C for 20 minutes. Sections were allowed to cool to room temperature and subsequently blocked in 10% normal goat serum with 1% bovine serum albumin in PBS for one hour. Slides were incubated at 4°C for one hour with monoclonal anti- PPP1R13L primary antibody (Sigma-Aldrich) at 1:200. Slides were then washed 3x in PBST and incubated for 20 minutes with goatαmouse Alexa633 (Invitrogen) secondary antibody at 1:1000. Slides were washed 3x in PBST and mounted with Vectashield mounting medium containing DAPI (Vector Labs).

### Linkage mapping

The *woe2* homozygote mice were outcrossed to C3A.BliA-*Pde6β*+/J; the resulting F1 progeny were backcrossed which generated 164 F2 progeny. These progeny were evaluated at three weeks of age for the presence of the wavy fur and were clinically evaluated for ocular abnormalities. Following phenotyping, the F2 progeny were euthanized and tissues were collected. Genomic DNA was purified from collected tissues as previously described [22]. Initially, 25 F2 progeny were genotyped as previously described [22] with polymorphic microsatellites from Chr. 6: *D6Mit188, D6Mit320, D6Mit323;* Chr. 7: *D7Mit340, D7Mit160, D7Mit148;* Chr. 11: *D11Mit19, D11Mit229, D11Mit294*; Chr. 12: *D12Mit12, D12Mit147, D12Mit52.* For fine mapping on chromosome 7 all 164 progeny were genotyped with *D7Mit1, D7Mit22, D7Mit224, D7Mit340, D7Mit160,* and *D7Mit148.* Linkage was determined using Map Manager QTX software (http://www.mapmanager.org).

### Sequence analysis of *Ppp1r13l*

For exonic and intron/exon junction genomic sequence analysis, primers were designed to anneal 50 bp upstream and downstream from intron/exon junctions. PCR products were amplified and sequenced as previously described [22]. For cDNA analysis, RT-PCR was performed using total RNA from wild-type, *woe2/+*, and *woe2/woe2* tissues, utilizing hearts, spleens, or eyes as previously described [22]. The list of primer sequences is summarized in Additional file 1: Table S1. Long range PCR was conducted using the GeneAmp XL PCR kit (Roche, Branchburg, NJ) with primers (5’ CTGTCCACAATTACAGAGGGATCTGAA G3’) and (5’ CGGAAAGACAGCTCATCCCCAAACTCT 3’) as previously described [22]. DNAStar software was utilized for comparative analysis of the sequences.

## Results

### The *woe2* phenotypes

**Figure 1:**
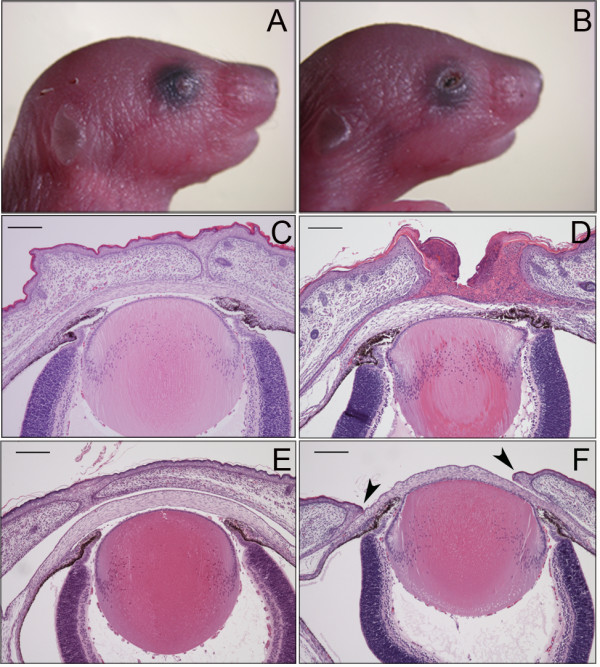
**The EOB phenotype in *woe2 *mice. **All *woe2 *neonates were born with bilateral open eyelids (**B**) in contrast to the WT neonates that have closed eyelids (**A**). Histological analysis of P0.5 *woe2 *mice confirmed the open eyelid phenotype and also revealed hyperkeratosis of the eyelids, stromal keratitis, absence of the conjunctival sacs, anterior synechiae, and hyperplastic corneas (**D**) features that were absent in the WT age matched controls (**C**). Further histological analysis of *woe2 *mice at E18.5 (**F**) showed failure of the embryonic eyelid closure (arrows) whereas WT controls exhibited embryonic eyelid closure (**E**).

At birth, *woe2* pups exhibit the fully penetrant, bilateral EOB phenotype (Figure 1B). Histological analysis of *woe2* eyes at P0.5 confirmed open eyelids at birth and also identified hyperkeratosis of the eyelids (Figure 1D). In addition to the eyelid defects, stromal keratitis, the absence of the conjunctival sacs, anterior synechiae, and hyperplastic corneas were also noted (Figure 1D). To determine if the EOB phenotype in *woe2* newborn pups was due to a defect in embryonic eyelid closure or due to premature eyelid opening at birth, we histologically evaluated E18.5 embryonic WT and *woe2* eyes. In WT mice, eyelid closure was completed between the two eyelids (Figure 1E), while in contrast, *woe2* mice exhibited open eyelids and exposed corneas (Figure 1F). This finding indicated that the EOB observed in the *woe2* newborn pups was due to the failure of embryonic eyelid closure.

**Figure 2:**
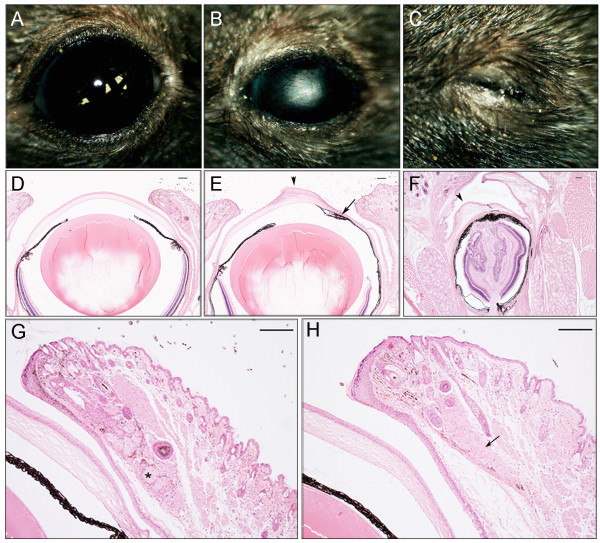
**The *woe2 *eye phenotype at P28. **Clinical evaluation of *woe2 *eyes identified microphthalmia with corneal opacities and neovascularization (**B) **when compared with WT mice (**A**). In about 25% of *woe2 *animals, unilateral or bilateral eyelid closure was noted (**C**). Histological analysis of *woe2 *eyes (**E**) identified anterior segment abnormalities with extensive anterior synechia (arrow), corneal hypertrophy (arrowhead) and stromal vascularization. Histological analysis of closed *woe2 *eyes (**C**) revealed aphakia with highly disorganized cornea, iris and retina (**F**). Histological analysis of adult *woe2 *eyelids (**H**) identified absence of meibomian glands (arrow*) *whereas meibomian glands were present in age-matched WT eyelids (**G**) (asterisk). Scale bar in D-H = 100 μm.

As *woe2* mice progressed through development, their eyes appeared smaller in size when compared to WT controls. To further investigate the *woe2* eye phenotype, we clinically evaluated P28 eyes. The analysis revealed opaque and vascularized corneas (Figure 2B) which precluded further clinical investigation. Clinical eye evaluation also identified that in about 25% of P28 *woe2* mice, unilateral or bilateral eyelid closure was present (Figure 2C). Histological analysis of P28 *woe2* eyes confirmed severely disrupted corneas and further identified anterior segment dysgenesis with extensive anterior synechiae, (Figure 2E) while no obvious morphological abnormalities of the lens and the retina were noted (not shown). Histological analysis of the *woe2* eyes which appeared closed (Figure 2C) revealed severely microphthalmic eyes as a result of aphakia (Figure 2F). In addition, highly disorganized corneal, iris and retinal structures were observed (Figure 2F). Histological analysis of P28 eye globes also revealed the absence of meibomian glands in the P28 *woe2* eyelids (Figure 2H).

**Figure 3:**
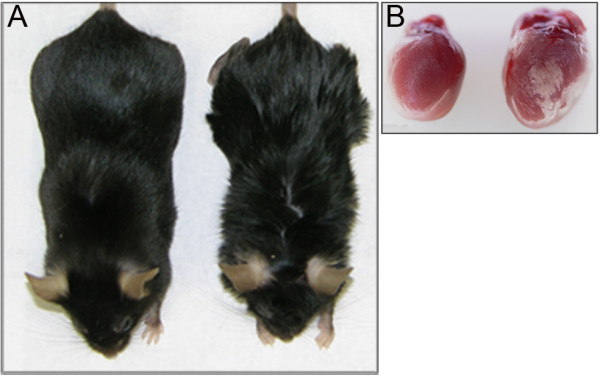
**Non-ocular *woe2 *phenotypes at P28. **The fur (**A**) in *woe2 *mice (right) showed waviness when compared to the smooth coat in WT mice (left). Hearts (**B**) of *woe2 *mice showed mineralized foci and opaque white plaques (right) that were absent in age-matched wild-type controls (left).

In addition to the ocular phenotypes, *woe2* mice start to exhibit wavy fur around P14 that remains wavy in texture throughout the life of the animal (Figure 3A). Abnormalities in the fur texture and the absence of meibomian glands identified in *woe2* eyelids (Figure 2H) prompted us to evaluate other epidermal appendages in *woe2.* Clinical and histological analysis of the skin, teeth, and nails as well as the lacrimal, mammary, salivary, sebaceous and sweat glands did not identify any obvious morphological differences between WT and *woe2* mice (not shown). However, the adult *woe2* mice exhibited cardiac abnormalities characterized with opaque white plaques covering approximately 2-15% of the exposed surface (Figure 3B). The mineralized foci tended to occur in the myocardium adjacent to the epicardium, although similar lesions occurred in the interventricular septum as well (not shown). On average, *woe2* mice lived until around 9 months of age indicating shortened life spans when compared to age-matched C57BL/6 J mice. The initial breeding pattern revealed that the *woe2* phenotypes were inherited as a fully penetrant single autosomal recessive locus.

### Positional cloning of *woe2*

**Figure 4:**
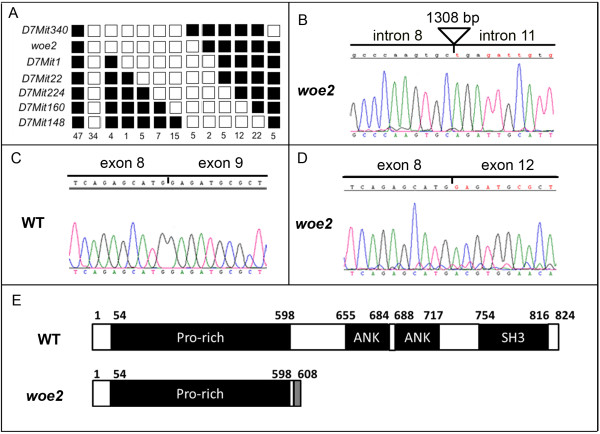
**The genetic analysis of the *woe2 *locus. **The *woe2 *locus was mapped to mouse chromosome 7 (**A**) between *D7Mit340 *and *D7Mit1 *establishing *Ppp1r13l *as a candidate gene. Each column represents the haplotype found in progeny of *woe2 *mutants (C57BLX129/SvJ background) when backcrossed to the C3A.BliAPde6+/J strain. Black boxes represent the C3ABliA-Pde6+/J allele and white boxes represent the C57BL6X129/SvJ allele. The number of offspring inheriting each haplotype is listed at the bottom of each column. Sequence analysis of genomic DNA from *woe2 *tissues (**B**) identified a 1308 bp deletion encompassing the genomic region from *Ppp1r13l *intron 8 to intron 11. RT-PCR analysis from WT tissues (**C**) identified a single *Ppp1r13l *transcript that matched the *Ppp1r13l *reference sequence (NM_001010836). RT-PCR analysis from *woe2 *tissues (**D**) identified a single aberrantly spliced *Ppp1r13l *transcript with missing exons 9, 10 and 11. (**E**) depicts a schematic of wild type PPP1R13L protein (NP_001010836) (top) conserved domains: Pro-rich domain (54–598), ANK1 domain (655–684), ANK2 (688–717), and SH3 (754–816) domain. The *woe2 *putative PPP1R13L^p.E602TX7^ protein (bottom figure) shows, following Met601, an addition of 7 novel amino acids (TWNKAWD) depicted by a gray box, a premature stop and a loss of 223 amino acids from the C-terminus encompassing ANK1, ANK2 and SH3 domains. Numbers on the top of each figure in (E) represent the amino acid residues.

The observed phenotypes of *woe2* mice resembled phenotypes of mice with mutations in *Tgfa, Egfr, Adam17* and *Ppp1r13l* genes [5-11,14,16]. Therefore, we hypothesized that *woe2* may be a new mutant allele in one of these four genes. To test that hypothesis, we utilized a genetic approach. *Tgfa, Ppp1r13l, Egfr,* and *Adam17* genes map to chromosomes 6, 7, 11 and 12 respectively (http://www.informatics.jax.org/). Therefore, we selected microsatellite markers from chromosomes 6, 7, 11 and 12 and tested segregation of the *woe2* locus with these microsatellites. No linkage was observed between the *woe2* locus and microsatellite markers on chromosomes 6, 11 and 12, but linkage of the *woe2* locus to chromosome 7 was established with LOD > 10. To refine the genetic position of the *woe2* locus we selected additional microsatellite markers from chromosome 7. The analysis mapped the *woe2* locus to a 6.8 cM region between *D7Mit340* and *D7Mit1* (Figure 4A). The established *woe2* critical region contained the *Ppp1r13l* gene. Therefore, we proceeded to evaluate *Ppp1r13l* as a candidate gene.

Genomic sequence analysis of *Ppp1r13l* exons 1–8 and exon 12 including intron/exon junctions did not identify any difference between the *Ppp1r13l* reference sequence (NC_000073.5) and sequences from *woe2*. Although conventional PCR produced PCR products for exons 9, 10, and 11 when using genomic DNA from WT animals, it did not produce any PCR products when using genomic DNA from *woe2* animals. This suggested a possible genomic rearrangement across the *Ppp1r13l* exon 9-11 region in the *woe2* animals. Long range-PCR from exon 8 to exon 12 and subsequent sequencing revealed a 1308 bp deletion in *woe2* (Figure 4B). The deletion break points were identified in *Ppp1r13l* intron 8 at −271 bp upstream from the 5' end of exon 9 and in intron 11 at −1203 bp upstream from the 5' end of exon 12 (Figure 4B).

To determine the molecular consequences of the 1308 bp deletion on the splicing of the *Ppp1r13l* transcript in the *woe2* tissues, we proceeded to evaluate *Ppp1r13l* cDNA. RT-PCR analysis from *woe2* tissues identified a single *Ppp1r13l* transcript that exhibited aberrant splicing from exon 8 to exon 12 with exons 9, 10 and 11 missing (Figure 4D). RT-PCR analysis of *Ppp1r13l* from WT tissues identified a transcript that matched the *Ppp1r13l* reference sequence (NM_001010836.3) (Figure 4C). The aberrant *Ppp1r13l*^*Δexon9-11*^ transcript identified in *woe2* encodes a putative PPP1R13L mutant protein with a frame shift following M601, an addition of 7 amino acids (TWNKAWD), and a premature stop. The putative mutant PPP1R13L lacks 223 amino acids from the C-terminal end (Figure 4E).

### The *woe2* EOB phenotype

**Figure 5:**
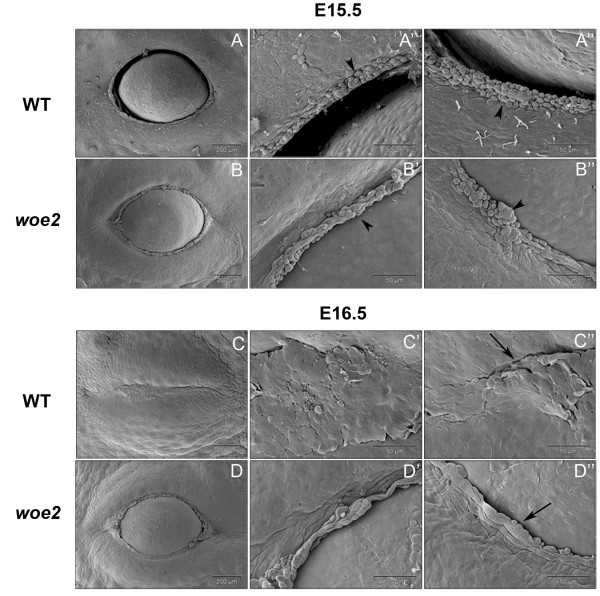
**Scanning electron micrographs showing impaired embryonic eyelid closure in *woe2 *embryos. **At E15.5 WT (**A, A', A"**) and *woe2 ***(B, B', B") **embryos manifested open eyes of similar shape and with no distinct morphological difference. The accumulation of rounded peridermal cells at the leading edge of both WT (**A', A"**) and *woe2 *(**B', B"**) eyelids was evident (arrowhead). Differences between the WT and *woe2 *eyelids were evident at E16.5. The eyelids of WT embryos were completely fused (**C, C', C"**) at the midline with flattened cells at the junction margins (**C” **arrow). In contrast, failure of eyelid closure was apparent in the eyes of *woe2 *mice at this time (**D, D', D"**). At this time point, very few peridermal cells were evident at the eyelid margin of *woe2 *eyelids (**D” **arrow).

To further investigate the embryonic eyelid closure defect that lead to the EOB phenotype in the *woe2* mice, we evaluated the embryonic eyelids with scanning electron microscopy (SEM) just prior to the embryonic eyelid closure. At E15.5, the leading edges were present in both WT and *woe2* upper (Figures 5A) and lower eyelids (Figure 5B). Higher magnification views showed presence of rounded periderm cells in both the WT and *woe2* eyelid margins (Figures 5A', 5A", 5B' and 5B"). At E16.5, in WT mice, the eyelid closure was completed (Figure 5C) and flattened cells were present at the eyelid junction (Figures 5C' and 5C"). By contrast, the *woe2* eyelids, at E16.5, remained wide open (Figure 5D). Rounded peridemal cells were infrequently seen at this stage at the leading edge of *woe2* (Figure 5D' and 5D").

### PPP1R13L expression in the developing eyelids

**Figure 6:**
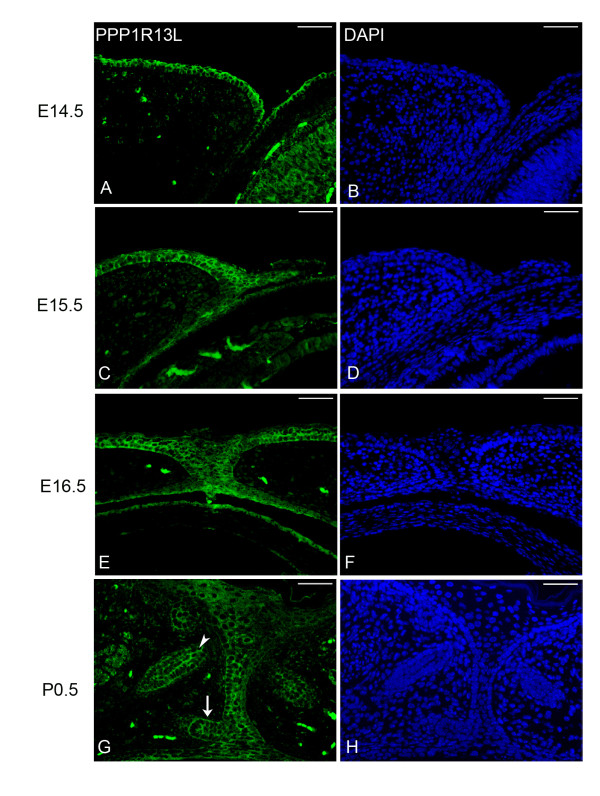
**PPP1R13L expression in the developing eyelid. **Immunostaining for PPP1R13L (green) showed expression of PPP1R13L at E14.5-P0.5 (**A, C, E, G**) in the palpebral epidermis, palpebral conjunctiva, bulbar conjunctiva, corneal epithelium, lens epithelium and retina. DAPI (blue) was used as a nuclear stain. As eyelid closure begins, at E15.5 (**C**) PPP1R13L is also expressed in the leading edge of the migrating eyelid. As eyelid closure reaches completion at E16.5 (**E**), expression is maintained in the eyelid junction. At P0.5 (**G**), PPP1R13L is expressed in the developing meibomian gland (**G** arrow) and hair follicle (**G** arrowhead). Scale bars = 50 μm.

Expression of PPP1R13L in mouse skin has been previously reported [16,19,20], although expression of PPP1R13L in the eyelids and anterior segment has never been investigated. In order to better understand the role of PPP1R13L during embryonic eyelid closure, the expression pattern of PPP1R13L during mouse eyelid closure was pursued. Immunohistological analysis showed PPP1R13L is expressed in the cells of the epithelial sheet of the developing eyelid including palpebral epidermis, palpebral and bulbar conjunctiva (Figure 6A and 6C). Following eyelid closure, PPP1R13L remained expressed in the cells of the eyelid junction (Figure 6E). In P0.5 eyelids, PPP1R13L was also identified as expressed in the developing hair follicle and in the cells of the developing meibomian glad (Figure 6G). In addition to the eyelid, immunohistological analysis identified expression of PPP1R13L in the corneal epithelium (Figure 6A and 6C), lens epithelium and the retina (not shown).

## Discussion

In this study we characterized *woe2*, a novel mouse mutation which arose spontaneously. Genetic analysis of the *woe2* locus identified a 1308 bp deletion in the *Ppp1r13l* gene encompassing exons 9 through 11 resulting in an aberrant *Ppp1r13l*^*Δexon9-11*^ transcript. The putative *woe2* PPP1R13L protein encoded by the *Ppp1r13l*^*Δexon9-11*^ transcript lacks 223 amino acids from the C-terminal end. PPP1R13L, at the C-terminal end, is characterized with ankyrin (AKN) repeats, and a SRC homology (SH3) domain [23]. The biochemical analyses of PPP1R13L have established that the ANK repeats and the SH3 domain are essential for the PPP1R13L binding with its partners: p53 [17], p65RelA [18], and p63 [24]. The 223 amino acids missing from the mutant PPP1R13L from *woe2* encompass both the ANK repeats and the SH3 domain. Although we did not functionally evaluate the truncated *woe2* PPP1R13L protein, we expect that a loss of 223 amino acids may compromise the stability of the mutant *woe2* PPP1R13L protein. Even if the mutant *woe2* PPP1R13L protein remains s, the loss of the ANK repeats and the SH3 domain most likely would result in a complete loss of PPP1R13L function. This is consistent with the findings previously reported for *wa3* mice that carry a 14-bp deletion in *Ppp1r13l* resulting in a loss of the SH3-domain from the C-terminal end [14]. The mutant PPP1R13L protein from *wa3* mice exhibited a loss of binding to the p65RelA subunit of NF-кB [14], a previously established PPP1R13L binding partner [16]. As such, *wa3* was established as a *Ppp1r13l* loss-of-function mutation [14]. These findings collectively suggest that *woe2* is a novel *Ppp1r13l* loss-of-function mutation.

The wavy fur and cardiac phenotypes identified in *woe2* resemble the cardiac and wavy fur phenotypes previously characterized in the *wa3* mice [14]. Abnormalities in the fur and heart were also reported for cattle affected with cardiomyopathy and wooly hair coats (CWH) syndrome that carry a frame-shift mutation in the bovine ortholog of the *Ppp1r13l* gene [25]. Although the EOB phenotype was also reported for *wa3* mice, it was not further investigated [14]. As a part of this study we established that in *woe2* the EOB phenotype is due to a defect in embryonic eyelid closure. Even though the morphological analysis of *woe2* embryonic eyelids did not identify any obvious abnormalities in the formation of the primitive eyelid, the formation of the leading edge or accumulation of peridermal cells at the leading edge, the *woe2* embryonic eyelids failed to close. Immunohistochemical analysis showed that PPP1R13L was expressed in the eyelid epithelial sheet including epidermis, palpebral and bulbar conjunctiva, as well as in the cells of the leading edge. It has been shown before that the embryonic eyelid closure process depends on the movements of the epithelial sheet of the developing eyelids in a morphogenic process similar to dorsal closure in *Drosophila *[25]. In the developing epidermis of the skin, via its interaction with p63, PPP1R13L regulates the expression of genes that play an essential role in cell-matrix adhesion, as well as in epithelial cell junctions [17]. In addition, PPP1R13L was identified to regulate cell senescence and is required for epithelial stratification [20]. Given that the role of PPP1R13L during embryonic eyelid closure has never been investigated, our current hypothesis is that PPP1R13L interacts with p63 in a similar way as it does in the developing epidermis of the skin and regulates the expression of the adhesion genes and/or genes involved in the differentiation of the cells of the leading edge. Our hypothesis is further supported by the observation that *p63*^*−/−*^ mice also exhibit the EOB phenotype even though the development of the eyelids proceeds normally prior to the embryonic eyelid closure [26]. Although our current hypothesis implicates a role of PPP1R13L via interactions with p63, we cannot exclude the possibility that PPP1R13L may also be involved in molecular processes via interactions with the two other partners p65RelA and/or p53. Therefore, the role of PPP1R13L, as well as which molecular pathways are affected during embryonic eyelid closure requires further investigation.

Results from this study also identified the absence of meibomian glands in the *woe2* eyelids. Meibomian glands along with hair, teeth, nails and exocrine glands are epidermal appendages formed from an ectodermal placode [27]. Ectodermal dysplasia syndromes comprise a group of greater than 175 highly diverse disorders characterized by abnormalities of at least two ectodermal tissues where at least one involves hair, teeth, nails or sweat glands. In ectodermal dysplasia patients, other structures exhibiting abnormalities may include the mammary glands, thyroid gland, thymus, anterior pituitary, adrenal medulla, central nervous system, external ear, melanocytes, cornea, conjunctiva, lacrimal gland and lacrimal duct and meibomian glands [28]. Interestingly, alterations of or the absence of meibomian glands was identified as one of the most reliable clinical ocular indications in ectodermal dysplasia patients [29]. In addition to the absence of meibomian glands, evaluation of *woe2* eyes revealed abnormalities of the cornea and the anterior structures. The corneal epithelium is a tissue also originating from the surface ectoderm [30]; proper maturation of the corneal epithelium plays an essential role in ocular development, especially for the development of the anterior segment [30]. Taken together, these findings, along with the structural hair abnormalities previously identified as responsible for the wavy fur in *wa3* mice [14], prompted us to morphologically evaluate other epidermal appendages in *woe2* mice. However, our analysis did not identify any obvious defects in teeth, nails, lacrimal, salivary, mammary, sebaceous and sweat glands in *woe2* mice. Our results suggest that PPP1R13L may have unique roles in the development of meibomian glands, cornea and hair follicles although PPP1R13L may have overlapping roles in the development of other ectodermal appendages.

It should be noted that the abnormalities in the cornea and anterior segment structures identified in *woe2* mice may be solely related to the premature exposure to the environment consequent to the failure of the embryonic eyelid closure. Furthermore, the absence of meibomian glands may be further contributing to the abnormalities of the cornea--especially the corneal epithelium. Meibomian glands produce a lipid-rich secretion called meibum that is released from the orifices of the glands; the meibum is spread across the ocular surface and mix with tears produced by lacrimal glands to produce tear film that covers the entire ocular surface and has a protective, lubricatory, nutritional, and antimicrobial roles [31]. A dysfunction of meibomian glands results in abnormalities of the tear film and consequently ocular irritation, inflammation and ocular surface disease [32]. At this point, the molecular etiology of the anterior segment defects in *woe2* remains unclear. It should be also noted that *woe2* EOB and ocular phenotypes reported in this study are very similar to phenotypes reported for mice with defects in EGFR signaling pathway [5-14]. While no functional relationship between PPP1R13L and EGFR signaling has been reported, the similarities in the eyelid and ocular phenotypes as well as hair phenotypes identified in *woe2* and mice with EGFR signaling defects suggest a common molecular pathway. Taken together, *woe2* mice provide an excellent resource for determining the role of PPP1R13L especially during development as well as for elucidating the molecular mechanisms associated with PPP1R13L function.

## Conclusions

The *woe2* mouse is a novel spontaneous autosomal recessive mutation exhibiting EOB and wavy fur phenotypes. Genetic analysis identified a deletion in the *Ppp1r13l* gene. A detailed morphological evaluation showed that EOB in *woe2* mice is due to a defect in embryonic eyelid closure. In addition to EOB, *woe2* mice exhibited defects in the eye anterior segment development and the absence of meibomian glands. The precise role of PPP1R13L during the embryonic eyelid closure, development of meibomian glands and anterior segment structures requires further investigation. We anticipate that *woe2* mice will provide a useful model in elucidating the etiology and underlying mechanisms of PPP1R13L function.

## Competing interests

The authors of this manuscript declare that they have no competing interests.

## Authors’ contributions

Design of the study, data analysis and critical revision of the manuscript was conducted by DJS and JAT. The original draft of the manuscript and experimentation was conducted by JAT. LL contributed to experimentation, analysis and interpretation of the data. This manuscript has been read and approved by all authors.

## Supplementary Material

Additional file 1 Sequences of PCR primers used in this study. Click here for file

## References

[B1] Findlater GS McDougall RD Kaufman MH Eyelid development, fusion and subsequent reopening in the mouse J Anat 1993 183 Pt 1 121 129 8270467 PMC1259860

[B2] Teraishi T Yoshioka M Electron-microscopic and immunohistochemical studies of eyelid reopening in the mouse Anat Embryol (Berl) 2001 204 2 101 107 10.1007/s004290100189 11556525

[B3] Li G Gustafson-Brown C Hanks SK Nason K Arbeit JM Pogliano K Wisdom RM Johnson RS c-Jun is essential for organization of the epidermal leading edge Dev Cell 2003 4 6 865 877 10.1016/S1534-5807(03)00159-X 12791271

[B4] Weng J Luo J Cheng X Jin C Zhou X Qu J Tu L Ai D Li D Wang J Deletion of G protein-coupled receptor 48 leads to ocular anterior segment dysgenesis (ASD) through down-regulation of Pitx2 Proc Natl Acad Sci U S A 2008 105 16 6081 6086 10.1073/pnas.0708257105 18424556 PMC2329706

[B5] Mann GB Fowler KJ Gabriel A Nice EC Williams RL Dunn AR Mice with a null mutation of the TGF alpha gene have abnormal skin architecture, wavy hair, and curly whiskers and often develop corneal inflammation Cell 1993 73 2 249 261 10.1016/0092-8674(93)90227-H 8477444

[B6] Luetteke NC Qiu TH Peiffer RL Oliver P Smithies O Lee DC TGF alpha deficiency results in hair follicle and eye abnormalities in targeted and waved-1 mice Cell 1993 73 2 263 278 10.1016/0092-8674(93)90228-I 8477445

[B7] Horiuchi K Kimura T Miyamoto T Takaishi H Okada Y Toyama Y Blobel CP Cutting edge: TNF-alpha-converting enzyme (TACE/ADAM17) inactivation in mouse myeloid cells prevents lethality from endotoxin shock J Immunol 2007 179 5 2686 2689 17709479 10.4049/jimmunol.179.5.2686

[B8] Hassemer EL Le Gall SM Liegel R McNally M Chang B Zeiss CJ Dubielzig RD Horiuchi K Kimura T Okada Y The waved with open eyelids (woe) locus is a hypomorphic mouse mutation in Adam17 Genetics 2010 185 1 245 255 10.1534/genetics.109.113167 20194968 PMC2870960

[B9] Peschon JJ Slack JL Reddy P Stocking KL Sunnarborg SW Lee DC Russell WE Castner BJ Johnson RS Fitzner JN An essential role for ectodomain shedding in mammalian development Science 1998 282 5392 1281 1284 9812885 10.1126/science.282.5392.1281

[B10] Luetteke NC Phillips HK Qiu TH Copeland NG Earp HS Jenkins NA Lee DC The mouse waved-2 phenotype results from a point mutation in the EGF receptor tyrosine kinase Genes Dev 1994 8 4 399 413 10.1101/gad.8.4.399 8125255

[B11] Threadgill DW Dlugosz AA Hansen LA Tennenbaum T Lichti U Yee D LaMantia C Mourton T Herrup K Harris RC Targeted disruption of mouse EGF receptor: effect of genetic background on mutant phenotype Science 1995 269 5221 230 234 10.1126/science.7618084 7618084

[B12] Miettinen PJ Berger JE Meneses J Phung Y Pedersen RA Werb Z Derynck R Epithelial immaturity and multiorgan failure in mice lacking epidermal growth factor receptor Nature 1995 376 6538 337 341 10.1038/376337a0 7630400

[B13] Du X Tabeta K Hoebe K Liu H Mann N Mudd S Crozat K Sovath S Gong X Beutler B Velvet, a dominant Egfr mutation that causes wavy hair and defective eyelid development in mice Genetics 2004 166 1 331 340 10.1534/genetics.166.1.331 15020428 PMC1470694

[B14] Lee D Cross SH Strunk KE Morgan JE Bailey CL Jackson IJ Threadgill DW Wa5 is a novel ENU-induced antimorphic allele of the epidermal growth factor receptor Mamm Genome 2004 15 7 525 536 15366372 10.1007/s00335-004-2384-2

[B15] Mine N Iwamoto R Mekada E HB-EGF promotes epithelial cell migration in eyelid development Development 2005 132 19 4317 4326 10.1242/dev.02030 16141218

[B16] Herron BJ Rao C Liu S Laprade L Richardson JA Olivieri E Semsarian C Millar SE Stubbs L Beier DR A mutation in NFkB interacting protein 1 results in cardiomyopathy and abnormal skin development in wa3 mice Hum Mol Genet 2005 14 5 667 677 10.1093/hmg/ddi063 15661756

[B17] Bergamaschi D Samuels Y O'Neil NJ Trigiante G Crook T Hsieh JK O'Connor DJ Zhong S Campargue I Tomlinson ML iASPP oncoprotein is a key inhibitor of p53 conserved from worm to human Nat Genet 2003 33 2 162 167 10.1038/ng1070 12524540

[B18] Yang JP Hori M Sanda T Okamoto T Identification of a novel inhibitor of nuclear factor-kappaB, RelA-associated inhibitor J Biol Chem 1999 274 22 15662 15670 10.1074/jbc.274.22.15662 10336463

[B19] Chikh A Matin RN Senatore V Hufbauer M Lavery D Raimondi C Ostano P Mello-Grand M Ghimenti C Bahta A iASPP/p63 autoregulatory feedback loop is required for the homeostasis of stratified epithelia EMBO J 2011 30 20 4261 4273 10.1038/emboj.2011.302 21897369 PMC3199390

[B20] Notari M Hu Y Koch S Lu M Ratnayaka I Zhong S Baer C Pagotto A Goldin R Salter V Inhibitor of apoptosis-stimulating protein of p53 (iASPP) prevents senescence and is required for epithelial stratification Proc Natl Acad Sci U S A 2011 108 40 16645 16650 10.1073/pnas.1102292108 21930934 PMC3189025

[B21] Liegel R Chang B Dubielzig R Sidjanin DJ Blind sterile 2 (bs2), a hypomorphic mutation in Agps, results in cataracts and male sterility in mice Mol Genet Metab 2011 103 1 51 59 10.1016/j.ymgme.2011.02.002 21353609 PMC3081956

[B22] Talamas E Jackson L Koeberl M Jackson T McElwee JL Hawes NL Chang B Jablonski MM Sidjanin DJ Early transposable element insertion in intron 9 of the Hsf4 gene results in autosomal recessive cataracts in lop11 and ldis1 mice Genomics 2006 88 1 44 51 10.1016/j.ygeno.2006.02.012 16595169 PMC1509100

[B23] Sullivan A Lu X ASPP: a new family of oncogenes and tumour suppressor genes Br J Cancer 2007 96 2 196 200 10.1038/sj.bjc.6603525 17211478 PMC2359998

[B24] Robinson RA Lu X Jones EY Siebold C Biochemical and structural studies of ASPP proteins reveal differential binding to p53, p63, and p73 Structure 2008 16 2 259 268 10.1016/j.str.2007.11.012 18275817

[B25] Simpson MA Cook RW Solanki P Patton MA Dennis JA Crosby AH A mutation in NFkappaB interacting protein 1 causes cardiomyopathy and woolly haircoat syndrome of Poll Hereford cattle Anim Genet 2009 40 1 42 46 10.1111/j.1365-2052.2008.01796.x 19016676

[B26] Shalom-Feuerstein R Lena AM Zhou H De La Forest Divonne S Van Bokhoven H Candi E Melino G Aberdam D DeltaNp63 is an ectodermal gatekeeper of epidermal morphogenesis Cell Death Differ 2011 18 5 887 896 10.1038/cdd.2010.159 21127502 PMC3131930

[B27] Mikkola ML Genetic basis of skin appendage development Semin Cell Dev Biol 2007 18 2 225 236 10.1016/j.semcdb.2007.01.007 17317239

[B28] Visinoni AF Lisboa-Costa T Pagnan NA Chautard-Freire-Maia EA Ectodermal dysplasias: clinical and molecular review Am J Med Genet A 2009 149A 9 1980 2002 10.1002/ajmg.a.32864 19681154

[B29] Kaercher T Ocular symptoms and signs in patients with ectodermal dysplasia syndromes Graefes Arch Clin Exp Ophthalmol 2004 242 6 495 500 10.1007/s00417-004-0868-0 14963716

[B30] Cvekl A Tamm ER Anterior eye development and ocular mesenchyme: new insights from mouse models and human diseases Bioessays 2004 26 4 374 386 10.1002/bies.20009 15057935 PMC2094210

[B31] Butovich IA The Meibomian puzzle: combining pieces together Prog Retin Eye Res 2009 28 6 483 498 10.1016/j.preteyeres.2009.07.002 19660571 PMC2783885

[B32] Nelson JD Shimazaki J Benitez-del-Castillo JM Craig JP McCulley JP Den S Foulks GN The international workshop on meibomian gland dysfunction: report of the definition and classification subcommittee Invest Ophthalmol Vis Sci 2011 52 4 1930 1937 10.1167/iovs.10-6997b 21450914 PMC3072158

